# An enhanced deep learning model for accurate classification of ovarian cancer from histopathological images

**DOI:** 10.1038/s41598-025-07903-9

**Published:** 2025-07-01

**Authors:** Anik Kumar Saha, Muntezar Rabbani, Anika Saba Ibte Sum, M. F. Mridha, Md Mohsin Kabir

**Affiliations:** 1 Department of Computer Science, American International University-Bangladesh, Dhaka, 1229 Bangladesh; 2 School of Innovation, Design and Engineering, Mälardalens University, 722 20 Västerås, Sweden

**Keywords:** Artificial Intelligence (AI), Convolutional Neural Networks (CNNs), Deep Learning, Ovarian Cancer (OC), Computational biology and bioinformatics, Nanoscience and technology

## Abstract

Ovarian Cancer is a malignancy that develops from ovarian cells and is frequently characterized by aberrant cell proliferation that leads to the creation of tumors within the ovaries. The high death rate and often delayed discovery of Ovarian Cancer make it a serious healthcare concern. Due to the annual 207,000 fatalities and 314,000 new cases worldwide, Ovarian Cancer poses a serious threat to public health, making quick and precise detection and classification techniques more essential. This work discusses the importance of Ovarian Cancer diagnosis and presents a new model for Ovarian Cancer classification. It also showcases a comparative analysis with other state-of-the-art models for Ovarian Cancer. Using an Ovarian Cancer image dataset which has data samples named Clear Cell, Endometri, Mucinous, Serous, and Non-Cancerous, it compares the proposed OvCan-FIND model to a wide range of CNN-based architectures, such as Inception V3, different EfficientNet variants, ResNet152V2, MobileNet, MobileNetV2, VGG16, VGG19, and Xception. The study examines the most recent Ovarian Cancer classification algorithms in this context to increase prognosis and diagnostic accuracy; our proposed OvCan-FIND model outperforms base models with an exceptional accuracy of 99.74%. This model presents significant prospects for enhancing ovarian cancer early identification and diagnosis, which will ultimately enhance patient outcomes.

## Introduction

Ovarian Cancer (OC), the eighth most common disease in women globally, poses a significant threat to women’s health due to its insidious nature, which is characterized by challenges in early detection, frequently resulting in a late-stage diagnosis with a poor prognosis^1^. OC is primarily made up of carcinomas, specifically epithelial-derived tumors, and has a diverse histological landscape that includes mucinous, clear cell, endometrioid, high-grade serous, and low-grade serous subtypes, as well as less common non-epithelial ovarian malignancies like mesenchymal, germ cell, and sex cord-stromal tumors. Notably, high-grade serous carcinoma accounts for 70% of all OC cases and has emerged as the most common subtype^2^. In the early stages, OC is asymptomatic^2^. It combined with an absence of efficient screening techniques presents a significant public health concern. OC is notoriously difficult to recognize and diagnose since it presents with ambiguous symptoms similar to menopausal symptoms. By the time symptoms appear, the disease has often progressed significantly, with primary malignant tumors extending throughout the abdomen, covering the ovaries, Fallopian tubes, and peritoneum (FIGO4 Stage 3)^1^. This delayed diagnosis contributes to an alarming mortality rate, with 314,000 new cases detected each year, resulting in 207,000 fatalities worldwide^1^. Precise classification of OC subtypes is crucial for developing individualized treatment strategies and addressing each subtype’s unique prognostic and therapeutic nuances. Several histological subtypes of the illness exist, the most prevalent of which are epithelial ovarian carcinomas^2^. Due to the unique morphological and prognostic features of these subtypes, accurate classification is required to provide patients with more effective treatment plans^2^. Though OC diagnosis is still best achieved by histopathological evaluation, which involves microscopic inspection of tissue specimens, Developing strong diagnostic tools is essential for early diagnosis and intervention of the malignancy, which is often detected at an advanced stage and is linked to a poor prognosis and high fatality rates. Automated categorization techniques are necessary to improve efficiency and accuracy because this procedure is labor-intensive, time-consuming, and susceptible to inter-observer variability. Automated categorization systems can speed up the diagnostic procedure, help identify OC early, and allow for individualized treatment plans based on histological subtypes. The need for computer-aided diagnosis methods to help interpret histopathological images and stratify OC cases according to subtype and stage is further highlighted by the fact that there is a global scarcity of pathologists. These devices can also enable medical professionals to provide prompt and accurate diagnoses in environments with limited resources, which will eventually improve patient care and survival rates. Advances in artificial intelligence and digital pathology have transformed the study of digitized whole slide images (WSIs) of ovarian tissue samples using automated classification approaches, notably those based on convolutional neural networks^3^. These sophisticated algorithms provide an appealing alternative to the difficulties involved with human histological investigation, demonstrating excellent accuracy in differentiating between distinct subtypes of OC. CNN-based models offer precise categorization by extracting complex features from histopathology images, which speeds up the diagnostic process and allows for early detection. Furthermore, these AI-driven systems have enormous potential for guiding personalized treatment regimens based on histological subtypes, hence improving patient care and results^3^. Thus, more research is a must for improved outcomes and better patient treatments.

Several recent studies have significantly advanced the field of OC prediction and treatment through the application of artificial intelligence methodologies. One such study^4^ conducted a comprehensive examination of OC prediction, focusing on the decision tree algorithm and machine learning techniques. The investigation introduced the MIDR (Machine Intelligent Doctor) model, designed to enhance accessibility to OC prediction, particularly for individuals with limited access to medical resources. Encouraging findings revealed the significant accuracy of the AI-based approach in OC classification, surpassing conventional prediction methods. Additionally, the study underscored the importance of scrutinizing factors such as dataset quality and generalizability across diverse populations to ensure the model’s reliability. Another pioneering study^2,5^ introduced a methodology for evaluating intratumoral cellularity in high-grade ovarian epithelial cancer, leveraging quantitative features derived from medical images and AI algorithms. The proposed technique achieved impressive accuracy in personalized OC treatment, emphasizing the potential of AI-driven methodologies in improving patient care. Furthermore, a study^5^ introduced a deep-learning AI framework utilizing histopathology images to anticipate OC responsiveness to platinum-based chemotherapy, demonstrating the model’s efficacy in accurately predicting treatment outcomes. Additionally, another study^6^ utilized a deep learning ensemble framework to predict treatment response in OC patients, achieving near-perfect prediction of patient response to bevacizumab treatment. Lastly, a study^2^ employed a deep learning approach to derive a signature from preoperative MRI data, facilitating the prediction of recurrence risk among patients diagnosed with high-grade serous OC. These studies collectively highlight the transformative potential of AI-driven strategies in advancing predictive healthcare solutions for OC diagnosis and treatment planning. The real-world applicability of current OC classification algorithms is hampered by problems with generalizability, overfitting, lack of clinical data integration, and dataset restrictions. Due to small and unbalanced datasets, many deep learning models currently in use suffer from inconsistent performance, which lowers classification task accuracy and reliability. These issues are addressed by our proposed OvCan-FIND model, which employs a task-specific deep-learning framework intended to reduce overfitting and improve resilience. Our methodology guarantees improved safety and accuracy in the categorization of histopathology images by streamlining the feature extraction and classification procedures. Furthermore, thorough tests against cutting-edge models and a variety of classification criteria confirm its exceptional performance, making it a more dependable and effective method for ovarian cancer screening. Even though there have been so much research on it, still there is always space for more accurate diagnosis and more improvements. So, our paper’s main purpose is to try to fill those gaps where more accurate results in OC classification can be achieved.

The creation and application of strong classification algorithms offer great potential for revolutionizing the field of OC diagnosis and treatment as research in these area progresses. The aim is to provide personalized patient care worldwide by enhancing the identification of OC through new insights obtained from this collaborative, multidisciplinary research initiative. So, the overall contributions of the analysis are:

- We proposed a task-specific advanced deep learning model for Ovarian Cancer Classification.
- Our strong and robust OvCan-FIND model is built for image classification by eliminating overfitting and performance issues.
- Enhancing safety and accuracy for Classification tasks on Histopathological images deploying the proposed model.
- We evaluated our proposed model with comparison of various state-of-the-art models and multiple classification task performance metrics.

Following the Introduction, the paper is structured as follows: In Section 2, the Literature Review has been presented. After that, Methodology was covered in Section 3. Followed by, Results Analysis in Section 4 and Discussion in Section 5. Finally, Section 6 presents the Conclusion.

## Literature review

Numerous latest studies have made significant strides in leveraging advanced technologies for the early detection, classification, diagnosis, and prognosis of OC. One study^6,7^ introduces a novel convolutional neural network algorithm tailored for predicting and diagnosing OC with impressive accuracy for classification. Trained on a histopathological image dataset, the CNN achieved a remarkable 94% accuracy rate, accurately identifying 95.12% of cancerous cases and 93.02% of healthy cells. However, limitations such as reliance on a public TCGA dataset, retrospective design, and absence of clinical data underscore the need for future research collaborations to validate the model on diverse patient populations and incorporate comprehensive clinical datasets to enhance its robustness and real-world applicability. Another study^8^ delves into prognostic indicators within pathological images of OC, utilizing a deep survival network. Leveraging the TCGA-OV dataset, the study employs meticulous preprocessing and segmentation techniques to predict hazard scores, aiding in stratifying cases into high and low-risk groups. Despite achieving a mean C-index value of 0.5789, indicating moderate prediction ability, the study highlights the potential of deep learning frameworks to offer clinically relevant prognostic insights in OC. Additionally, a paper^9^ presents a novel Deep Semi-Supervised Generative Learning with Enhanced U-Net and fused Deep Convolutional Neural Network (DSSGL-EUNet-DCNN) model for segmenting and classifying OC from CT scans. Experimental results demonstrate that the proposed model outperforms traditional approaches, reducing training errors and time costs while achieving higher accuracy in segmentation and classification tasks. Furthermore, another innovative technique^10^ introduces optical photothermal infrared (O-PTIR) imaging coupled with deep learning to enhance spatial resolution and enable sub-cellular spectroscopic examination of tissue for OC diagnosis and classification. Leveraging a dataset comprising patient samples, the approach achieves a high classification accuracy for ovarian cell subtypes and proposes quantitative biomarkers for early cancer detection and classification. Lastly, a comprehensive examination of several machine learning algorithms used in ovarian tumor classification^11^ highlights the efficiency of models like the C5.0 in predicting OC recurrence and recognizes CNNs as particularly effective for tumor diagnosis. While machine learning techniques show promise in improving accuracy compared to standard statistical methods, the study acknowledges limitations in discussing algorithm shortcomings and potential data quality difficulties, as well as constraints in ultrasound imaging, such as examiner-dependency and outcome variability. Furthermore, the study^12^ achieves a 100% accuracy improvement in cancer subtype classification by employing Support Vector Machine (SVM) and Cat Swarm Optimization (CSO) for feature selection. High processing requirements, the possibility of overfitting, and the requirement for more extensive validation on a variety of datasets are some of the drawbacks. Wavelet scattering transform (WST) and YOLO-based deep learning approaches have been used in recent studies to construct automated computer vision models for diagnosing dengue from peripheral blood smear (PBS) pictures^13^. Notwithstanding issues with dataset specificity, the WST approach and YOLOv8 models demonstrated the potential for early dengue detection and their applicability to other mosquito-borne diseases, achieving a classification accuracy of 98.7% with support vector machines and a mean accuracy of 99.3%, respectively^13^. In a similar vein, another study presented a deep learning approach for the detection of Alzheimer’s disease (AD) using T1-weighted MRI images. Using a volumetric convolutional neural network (ConvNet) that was improved by preprocessing and augmentation techniques, the study achieved 97% accuracy in differentiating AD from normal controls (NC)^14^. In order to advance automated AD diagnosis, this study addressed the difficulties posed by small datasets and highlighted the crucial importance of high-resolution MRI scans^14^. Additionally, by comparing GoogLeNet, ResNet18, and ResNet50, the identification of sickle cell disease (SCD) utilising deep neural networks and explainable artificial intelligence (XAI) was investigated; the best accuracy of 94.90% was attained by ResNet50^15^. In addition to outlining future strategies to address dataset generality, class imbalance, and ethical challenges in AI-driven healthcare solutions, the work used Grad-CAM and transfer learning to improve classification accuracy and interpretability, providing pathologists with significant benefits^15^.

Moreover, recent research has showcased a myriad of innovative approaches aimed at enhancing the detection, classification, and diagnosis of ovarian tumors, leveraging cutting-edge technologies such as deep learning and machine learning. For instance, one study^16^ proposes an advanced methodology utilizing the Anisotropic Diffusion Filter (ADF) for preprocessing, Improved Whale Search Optimization (IWSO) for segmentation, and Deep Neural Network (DNN) for classification in ultrasound images. The approach demonstrates superior performance across various metrics, including sensitivity, specificity, accuracy, and error rates, making it a promising technique for clinical use. This study^17^ analyzes AI techniques in omics data processing, highlighting their application in precision therapy, biomarker discovery, and disease classification. A small sample size, difficulties with data preprocessing, and the requirement for additional AI algorithm tuning are some of the limitations, too. Another investigation^18^ delves into machine learning approaches for accurately classifying benign ovarian tumors and OC, highlighting the significance of feature selection in improving model performance. While Random Forest with feature selection obtains the highest accuracy, deep learning approaches show comparable results, albeit with limitations such as dataset reliance on Kaggle and lack of detailed methodology information. Additionally, a study^19^ focuses on predicting OC types using classifiers like Support Vector Machine (SVM), Random Forest, and XGBoost. Despite challenges posed by an imbalanced dataset, the research underscores the need for more robust preprocessing methods and feature engineering techniques. Furthermore, another investigation^20^ introduces OCCNet, an Ensemble Attention Mechanism (EAM) designed to handle imbalanced datasets and categorize OC subtypes. The study demonstrates the model’s excellent subtype identification capabilities, albeit acknowledging the necessity for further fine-tuning and data augmentation to improve consistency. Moreover, a pioneering attempt^21^ utilizes the VGG-16 deep convolutional neural network architecture for automatic detection and classification of OC subtypes from histopathology images, showcasing its effectiveness despite dataset size constraints. Meanwhile, a novel transformer-based deep learning network^22^ demonstrates remarkable performance in detecting OC, yet issues regarding model generalizability and real-world applicability warrant further investigation. Additionally, a study^23^ explores the efficacy of deep learning models in distinguishing between malignant and non-cancerous ovarian histopathology images, with VGG-19 emerging as the top-performing model. Furthermore, an automated MRI analysis technique^24^ utilizing deep convolutional neural networks (DCNNs) shows promise in improving diagnostic precision, albeit requiring further validation in real-world clinical settings. Lastly, a unique strategy^25^ combining CNNs with Grey Wolf Optimization (GWO) demonstrates high accuracy in early OC diagnosis, while Ocys-Net^26^ showcases promising results in accurately classifying and diagnosing ovarian cysts, highlighting its potential for clinical applications.

Additionally, another paper^11^ addressed a wide range of methods used in OC research, from deep learning models for tumor segmentation and classification to preprocessing methods for CT scan images. The importance of deep learning approaches in medical image processing has been highlighted by the use of various architectures for classification, segmentation, and detection, including CNN, ResNet, DeepLabv3, TransUnet, and UNet, for a variety of applications^3,27–29^. Furthermore, high accuracy in tumor localization and segmentation has been shown by hybrid approaches combining detection and segmentation models, such as the attention U-Net segmentation model and the YOLO v5 detection model^30^. It highlights the significance of subtype-specific research in improving treatment outcomes. Furthermore, developments in model development and physician involvement suggest that computer-aided diagnostics (CAD) based on machine learning holds the potential for objective tumor assessment. Nevertheless, issues like overfitting and the requirement for bigger datasets as well as outside validation continue to exist^31^. However, there is still room for improvement due to constraints such as small dataset sizes and potential bias from manual segmentation. In the meantime, the integration of radiomics characteristics with deep learning has sought to increase tumor classification accuracy, sensitivity, and specificity^32^. In addition, research on molecular pathways and predictive models, such as evaluating the NHEJ pathway and forecasting treatment response, provides information on possible therapeutic targets and individualized treatment plans^33,34^. An extensive analysis of AI techniques used on histopathology images emphasizes the significance of physician participation and open reporting. Future developments include strong validations and better accessibility of data^35^. A thorough assessment of AI algorithms applied to histopathology images emphasizes the significance of clinician involvement and transparent presentation of data and code for enhancing therapeutic value. Furthermore, it has been demonstrated that combining multiomics data with artificial intelligence can improve model performance; in particular, data integration can help address issues like inter-observer variability and data scarcity that arise from manual segmentation^36^.

Table 1 summarizes and compares the existing research methods, their results and their limitations. The potential for deep learning models to improve diagnostic accuracy and offer prognostic insights that are pertinent to clinical settings. Notwithstanding encouraging outcomes, issues including overfitting, dataset dependence, and the requirement for more extensive, varied datasets and thorough clinical data continue. Furthermore, the use of artificial intelligence (AI) and deep learning techniques in OC research is the main emphasis of this literature review, which covers a wide range of novel approaches and methodologies. Studies have looked into a variety of AI models for tasks like tumor segmentation, classification, treatment response prediction, and prognosis evaluation. These models include convolutional neural networks, decision tree algorithms, and deep learning frameworks. The considerable accuracy attained by AI-driven methods for OC diagnosis, prognosis, and treatment planning are among the important discoveries. Yet, it can not be said that the results so far achieved are the best possible results or can not be improved further. Our research will prove to create a better version of the previous ones, and can help in achieving better results. In summary, the review highlights how AI-driven approaches can revolutionize the field of predictive healthcare for OC.

**Table 1:** Summary of few recent works on Ovarian Cancer identification methods, their results and Limitations.

**Ref.**	**Year**	**Dataset**	**Methods**	**Result**	**Limitations**
^37^	2024	UBC Ovarian Cancer Subtype Classification and Outlier Detection (UBC-OCEAN)	By combining encoder and decoder blocks with an integrated attention layer, the study created a novel model called Attention Embedder.	Testing accuracy on independent images was 91.37% at 40$\times$ magnification and 93.56% at 20$\times$ magnification.	The analysis faced difficulties with image variability, a sizable dataset (550 GB), and data imbalance (EC subtype elimination), all of which had an effect on efficiency, error handling, and practical application.
^38^	2024	Histopathological whole slide images for classification of Treatment effectiveness to ovarian cancer	To improve classification accuracy, the study employed a fuzzy deep learning classifier, recursive feature elimination for feature selection, ResNet50 for feature extraction, and data augmentation.	The suggested model distinguished between ovarian cancer and non-cancer images with an impressive 98.99% accuracy rate. It also showed an F1-score of 98.99%, a sensitivity of 99%, and a specificity of 98.96%.	The model’s effectiveness and ability to be generalized may be impacted by the study’s small dataset of 288 WSIs, particular augmentation methods, and unproven heterogeneity in histopathological pictures and staining.
^39^	2024	Collected and issued by third parties, namely the Dutch pathology registry (Palga) and the Netherlands Cancer Registry (NCR)	The work improved the histotyping accuracy of epithelial ovarian cancer (EOC) by using immunohistochemical decision-tree algorithms. The two primary algorithms that were created were the four-split algorithm (4S-alg) and the six-split algorithm (6S-alg).	The precision of the six-split method was 96.1%, whereas the precision of the four-split and six-split-stages algorithms was 93.5%.	Variable algorithm sensitivity was discovered in the study, along with difficulties in differentiating certain histotypes and restrictions brought on by the use of immunohistochemical markers, which impacted classification accuracy.
^40^	2024	Not specified	To improve the identification of ovarian cancer cells, the study employed a hierarchical classification method using label-free QPI-FC, retrieved 44 phase and texture data, and applied various machine learning models.	With a recall of 95.3% for OCCs and 91.8% for WBCs, the technique effectively separated OCCs from WBCs, demonstrating the usefulness of the QPI-FC methodology in a liquid biopsy setting.	Low cell numbers and OCC heterogeneity were difficulties for the study, which also recognized the possibility of increasing categorization accuracy through the use of cutting-edge AI methods like deep learning.
^41^	2024	Multi-parametric magnetic resonance imaging (mpMRI) data	With a 30% likelihood of data augmentation and a dynamic learning rate adjustment to maximize performance, the study presented a deep learning model for ovarian cancer classification with mpMRI data fusion.	With an F1 score of 0.8933 and an average accuracy of 86.3%, the model demonstrated its effectiveness in sample classification.	Additional refinement is required for clinical deployment, the study’s sample size restricts applicability, and the absence of benchmarking makes it difficult to compare results.

## Methodology

**Fig. 1:**
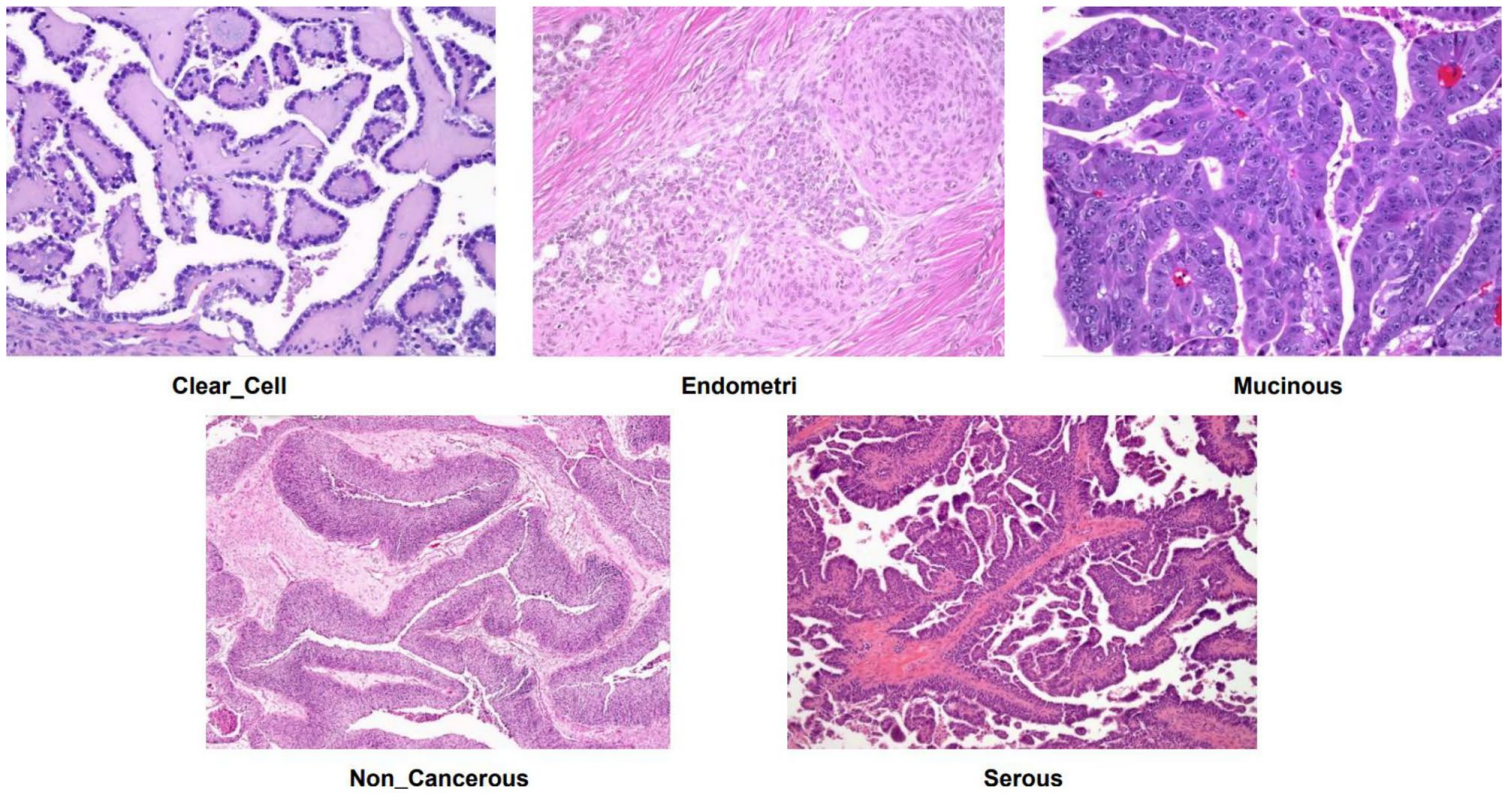
Histopathology sample images.

The sources, sample standards, and any filtering methods employed for ensuring high-quality inputs for the model are all described in this section of the study, which also covers the data collection approach and sample data shown in Figure 1. Normalization, augmentation, and data splitting all crucial for enhancing model robustness and minimizing overfitting are covered in detail, as are other steps in the data pre-processing workflow. The development of the suggested model is also discussed, emphasizing the architecture’s distinctive design and the choice of evaluation metrics including accuracy, precision, recall, and F1 score to thoroughly evaluate the model’s classification performance.

### General overview of the method

The categorization of OC through the comparison of different CNN models is the main emphasis of this study. The first phases in the method’s data pre-processing include making sure files are accessible, converting images to JPG format, and shrinking and padding them to standardize their size. To split a dataset, separate it into training, validation, and testing sets. To improve model generalization and boost dataset diversity, augmentation techniques such as brightness modification, rescaling, flipping, random rotation, and translation are used. The proposed model with preloaded ImageNet weights serves as the model architecture. Using the Functional API, more layers are added after removing the top layer. These layers include global average pooling, dropout layers for regularization, dense layers with ReLU activation and kernel regularization, and a final dense layer with softmax activation for multi-class prediction. To fine-tune the system to new weights, the first 100 layers are frozen, and then the layers after that are unfrozen. For multi-class classification, a categorical cross-entropy loss function and an Adam optimizer are used. To minimize overfitting and maximize model training, early stopping functions and learning rate scheduling are used. In general, the goal of this approach is to efficiently classify OC through the use of deep learning techniques, all the while maximizing model performance through architectural tweaks and data augmentation.

### Dataset description

The dataset used in this research consists of 85 images that were taken from 42 patients who were receiving care at Smt. Kashibai Navale Medical College and General Hospital in Pune, India^42^. Interestingly, there are no malignant cells in these images, which poses a problem because there aren’t many people in the hospital’s database who have been treated for OC (OC) other than serous carcinoma. Competent pathologists made it easier to get patient samples and prepare slides in their lab thereafter. Staining cell samples with a Leica ICC50 microscopic camera allowed for the real-time transfer of high-definition images to laptops and smartphones. Furthermore, images of carcinomas indicative of each OC subtype were obtained from The Cancer Repository, an open-access resource that requires registration. As a preprocessing step, the RGB images were uniformly resized to ensure compliance with state-of-the-art (SOTA) models for OC prediction. images of carcinomas linked with OC, including malignant tumors across four different subtypes, are included in the dataset along with images of normal tissues and benign images of glandular tissues that are not tumors. With a focus on the subtypes classified as carcinomas, the main goal of this dataset is to make it easier to classify OC automatically using histopathological images. These four subtypes’ example images also non-cancerous histopathological images are displayed in Figure 1. The image distribution for the various classes’ trains, tests, and validations is also shown in Table 2. For training, validation, and testing, the images were divided into 80%−10%−10% ratios, respectively.

**Table 2:** Image dataset distribution for each class.

**Class Name**	**Train**	**Validation**	**Test**	**Total Images**
Clear Cell	956	120	120	1196
Endometri	940	235	235	1175
Mucinous	718	239	239	1196
Serous	706	235	235	1176
Non Cancerous	719	240	240	1199
**Total**	**3566**	**1188**	**1188**	**5942**

### Data pre-processing

In order to increase our model’s resilience and versatility, we include many crucial phases in the image dataset preparation procedure. We initially utilize picture normalization to normalize pixel values so that our neural network’s input scale remains consistent. Following the normalization procedure, we incorporate data augmentation methods to enhance our dataset and lessen the likelihood of overfitting. Applying adjustments including converting images to JPG format, resizing and resizing them to a uniform size, rotating, flipping, zooming in or out, and moving them are all part of this approach. These augmentations increase the variety of our training data by improving the model’s capacity to handle distortions and variations in real-world images. The purpose of performing these preprocessing steps is to create a more robust and flexible model that can learn and generalize from the given data in an efficient manner, improving its performance on unknown images.

### Proposed model

**Fig. 2:**
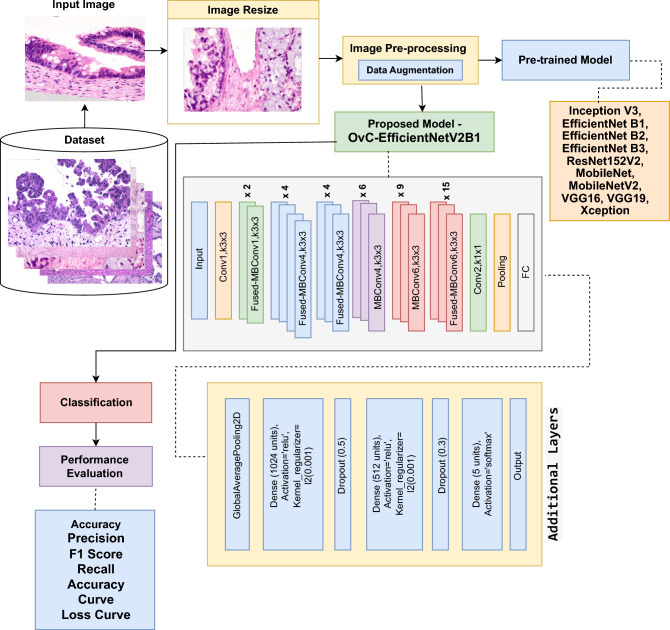
Overview of proposed OvCan-FIND system’s architecture.

The benchmarking models are trained using the histopathology image dataset, as seen in Figure 2. To guarantee data consistency and accessibility, pre-processing procedures included resizing images, converting files to a common format, and verifying file accessibility. By adding modifications to the training data, data augmentation techniques such as random rotation, translation, flipping, zooming, and brightness alterations were used to improve the robustness and generalization of the model. To enable model evaluation and performance assessment, the dataset was divided into training, validation, and testing sets according to a standard ratio. Effective classification tasks were made possible by utilizing the state of the art base model, which had been pre-trained on ImageNet weights. This allowed us to have access to an effective standardized benchmarking method and protocol with resources for classification evaluation.

Our proposed model, OvCan-FIND, is designed with layers specifically aimed at enhancing classification accuracy. To condense feature maps, we implemented techniques that streamline the data. We then added layers to extract higher-level features and incorporated methods to prevent overfitting. By randomly deactivating certain neurons during training, we reduced the risk of overfitting and improved the model’s ability to generalize. Finally, the model is capable of predicting the likelihood that an image belongs to one of the five ovarian cancer classifications, ensuring accurate and reliable results.

The reason for selecting the EfficientNetV2B1 model as our base is that Tan and Le (2021) have shown that EfficientNetV2B1 balances accuracy and efficiency, making it a good choice for cancer classification on moderate-sized datasets (thousands to tens of thousands of images)^43^. Because of its compact architecture, which lowers computational needs, it performs well even on GPUs with low memory^43^. Faster training and inference are also made possible by its optimized design, which makes it a viable option for real-time clinical applications where prompt diagnosis is essential^44^. EfficientNetV2B1’s efficacy in medical imaging applications is further supported by its excellent accuracy in picture categorization tests^44^.

So, building on the EfficientNetV2B1 architecture, the OvCan-FIND model incorporates domain-specific improvements for the classification of ovarian cancer while utilizing its improved feature extraction capabilities. Depthwise separable convolutions, which preserve high representational capacity while lowering computing complexity, are incorporated into the model. The network’s capacity to recalibrate feature maps is further improved by the addition of squeeze-and-excitation (SE) blocks, which highlight important patterns that are essential for distinguishing between subtypes of ovarian cancer. Batch normalization is also used at various stages to reduce internal covariate shifts, speed up convergence, and stabilize learning. The model uses a hybrid activation method, using ReLU in the initial layers for effective feature extraction and the Swish activation function in deeper layers for smooth gradient flow. To increase robustness against fluctuations in histopathology pictures, a progressive resizing method is also used, in which images go through multi-scale training. OvCan-FIND is optimized for high generalization performance by fusing these cutting-edge methods with meticulous data augmentation and regularization, guaranteeing accurate classification results across a variety of datasets.

The convolutional neural network model OvCan-FIND was trained with hyperparameters that reveal information about the optimal configuration for classifying OC which is given in Table 3. The model architecture has six layers and roughly 6.93 million parameters. The first three layers are 3x3 convolutional filters, while the next three are fully linked layers in the head. Preprocessing causes the images to be scaled to (625, 450), and the training data to be standardized to a resolution of (224, 224) with three channels. The Adam optimizer trains the model, with an adjustable learning rate set at 0.0001. The ReduceLROnPlateau scheduler and EarlyStopping method are used to control learning. The categorical CrossEntropy loss function is used for multi-class classification, with a label smoothing default of 0.1. To improve data diversity, augmentation techniques like rotation, flipping, normalization, and zooming are used. L2 kernel regularization and dropout rates of 0.5 and 0.3 are examples of regularization techniques. The network uses the activation functions Swish and ReLU in different places. Class prediction for the five OC classes is made possible by the classifier’s use of the softmax function to generate probabilities across a maximum of 30 epochs during training, using batches of size 32.

**Table 3:** Hyper Parameters for the proposed OvCan-FIND model.

**Hyper Parameters**	**Hyper Parameter Value**
Model	OvCan-FIND
Params	6.93 million
Convolution Size	3x3
Head	3
Layers	6
Training Resolution	(224, 224)
Number of Channels	3
Number of Classes	5
Scaling	Resize (625, 450)
Optimizer	Adam
Learning Rate	Adaptive, 0.0001 (at beginning)
Scheduler	ReduceLROnPlateau, EarlyStopping
Loss	Categorical CrossEntropy
Label Smoothing	0.1 by Default
Augmentation	Normalization, Rotation, Flipping, Zooming
Regularization	Dropout = 0.5, 0.3 | Kernel Regularizer = L2
Activation	Swish, ReLU
Max Epoch	30
Batch	32
Classifier	Softmax

## Result analysis

This section outlines the hardware and software specs, libraries, and frameworks utilized to achieve optimal performance and reproducibility in the environmental setup for the suggested model. The results research comprises a thorough assessment of the model’s outputs, contrasting its classification performance with that of other designs and benchmarks. To evaluate the performance of our implemented models and compare them with our proposed OvCan-FIND, we used several evaluation metrics, such as Accuracy (Acc), Precision (Pre), Recall (Rec), and F1 Score. In addition, we produced a confusion matrix, which is the source of extra metrics, while not strictly speaking a performance indicator. The confusion matrix shows the difference between the projected labels and the ground truth labels visually. Whereas columns in the confusion matrix describe cases in an actual class, rows in the matrix define examples in To class. There are terms that depend on the confusion matrix: False Positive (FP), False Negative (FN), True Positive (TP), and True Negative (TN).

A thorough explanation of the mathematical intuitions behind the following is provided below: Precision (Pre), Recall (Rec), Accuracy (Acc), and F1 Score, where-

*TP* = The model correctly predicted a number of positive class samples.

*TN* = The model predicted a number of negative class samples with accuracy.

*FP* = The model incorrectly predicted a number of negative class samples.

*FN* = The model failed to accurately forecast a number of positive class samples.

Accuracy (Acc) = $$\frac{T P+T N}{T P+T N+F P+F N}$$

Precision (Pre) = $$\frac{T P}{T P+F P}$$

Recall (Rec) = $$\frac{T P}{T P+F N}$$

F1 Score = $$\frac{2 \times \text{ Precision } \times \text{ Recall } }{ \text{ Precision } + \text{ Recall } }$$

Overall, the study presents a comprehensive approach to improving OC using seven models, including Inception V3, EfficientNet B1, EfficientNet B2, EfficientNet B3, ResNet152V2, MobileNet, MobileNetV2, VGG16, VGG19, and Xception, which are Convolutional Neural Networks. In order to enhance representation using the dataset, the research focuses on merging OvCan-FIND with extra layers. The suggested approach uses deep learning on the image dataset, and it highlights the hyperparameter configuration for OvCan-FIND for the classification of OC.

### Environment setup

Table 4 contains a thorough summary of the environment configuration used to train different CNN models. Table rows are associated with individual models and include key information like Model Name, GPU Name, Batch Size, Optimizer, Learning Rate, Epoch, Activation, and Data Augmentation.

**Table 4:** The environment setup summary for the models’ implementations.

**Model name**	**GPU name**	**Batch size**	**Optimizer, Learning rate**	**Epoch**	**Activation function**	**Data augmentation**
EfficientNetB1	NVIDIA TESLA T4	224x224	Adam, lr:0.0001	30	ReLU	Not Applied
EfficientNetB2	NVIDIA TESLA T4	224x224	Adam, lr:0.0001	30	ReLU	Not Applied
EfficientNetB3	NVIDIA TESLA T4	224x224	Adam, lr:0.0001	30	ReLU	Not Applied
InceptionV3	NVIDIA TESLA T4	224x224	Adam, lr:0.0001	30	ReLU	Not Applied
MobileNet	NVIDIA TESLA T4	224x224	Adam, lr:0.0001	30	ReLU	Not Applied
MobileNetV2	NVIDIA TESLA T4	224x224	Adam, lr:0.0001	30	ReLU	Not Applied
ResNet152V2	NVIDIA TESLA T4	224x224	Adam, lr:0.0001	30	ReLU	Not Applied
VGG16	NVIDIA TESLA T4	224x224	Adam, lr:0.0001	30	ReLU	Not Applied
VGG19	NVIDIA TESLA T4	224x224	Adam, lr:0.0001	30	ReLU	Not Applied
Xception	NVIDIA TESLA T4	224x224	Adam, lr:0.0001	30	ReLU	Not Applied
Proposed Model (OvCan-FIND)	NVIDIA TESLA T4	224x224	Adam, lr:0.0001	30	ReLU	Applied

Interestingly, a maximum of 30 epochs and a consistent batch size of 224x224 were used to train all models on the NVIDIA TESLA T4 GPU. To add non-linearity to the network, the activation function ReLU was applied consistently to every model. Although some models used the Adam optimizer with a fixed learning rate of 0.0001, the OvCan-FIND model that was proposed used Adam with an adaptive learning rate that starts at 0.0001. Interestingly, data augmentation was only used in the proposed model’s training phase, with the goal of incorporating random modifications to improve the model’s robustness and generalization performance. This configuration facilitates reproducibility and comparison across many experiments and architectures by offering important insights into the typical setting for CNN model training in the context of OC classification.

### Result analysis

By comparing the performance of different models with histopathology data on an OC subtype dataset in Table 5, significant differences in accuracy metrics were found. EfficientNetB1, B2, and B3 showed reasonable validation accuracies between 0.67 and 0.79 and corresponding testing accuracies between 0.71 and 0.83 across the evaluated models. On the other hand, MobileNetV2 performed noticeably worse on all criteria, suggesting difficulties in correctly identifying OC subtypes. ResNet152V2 performed well compared to the other models examined, displaying competitive results with a testing accuracy of 0.83 and a validation accuracy of 0.75. OvC-EfficientNetV281, the suggested model, fared better than all the others, with testing and validation accuracies of 0.9974 and 0.9933, respectively. This outstanding result highlights the effectiveness of the suggested methodology and demonstrates how well it can classify OC subtypes based on the histopathology dataset.

**Table 5:** Model Accuracy Comparison for OC Classification using Subtypes Histopathology dataset with proposed OvCan-FIND model.

**Model name**	**Validation accuracy**	**Precision**	**Recall**	**F1 score**	**Testing accuracy**
EfficientNetB1	0.79	0.69	0.69	0.67	0.75
EfficientNetB2	0.79	0.80	0.80	0.79	0.75
EfficientNetB3	0.79	0.76	0.78	0.76	0.83
InceptionV3	0.67	0.77	0.75	0.75	0.79
MobileNet	0.79	0.73	0.74	0.73	0.71
MobileNetV2	0.38	0.23	0.24	0.12	0.25
ResNet152V2	0.75	0.74	0.74	0.74	0.83
VGG16	0.67	0.62	0.63	0.61	0.66
VGG19	0.58	0.61	0.56	0.54	0.58
Xception	0.79	0.80	0.80	0.78	0.79
Proposed Model (OvCan-FIND)	0.9933	0.9968	0.9956	0.99	0.9974

**Fig. 3:**
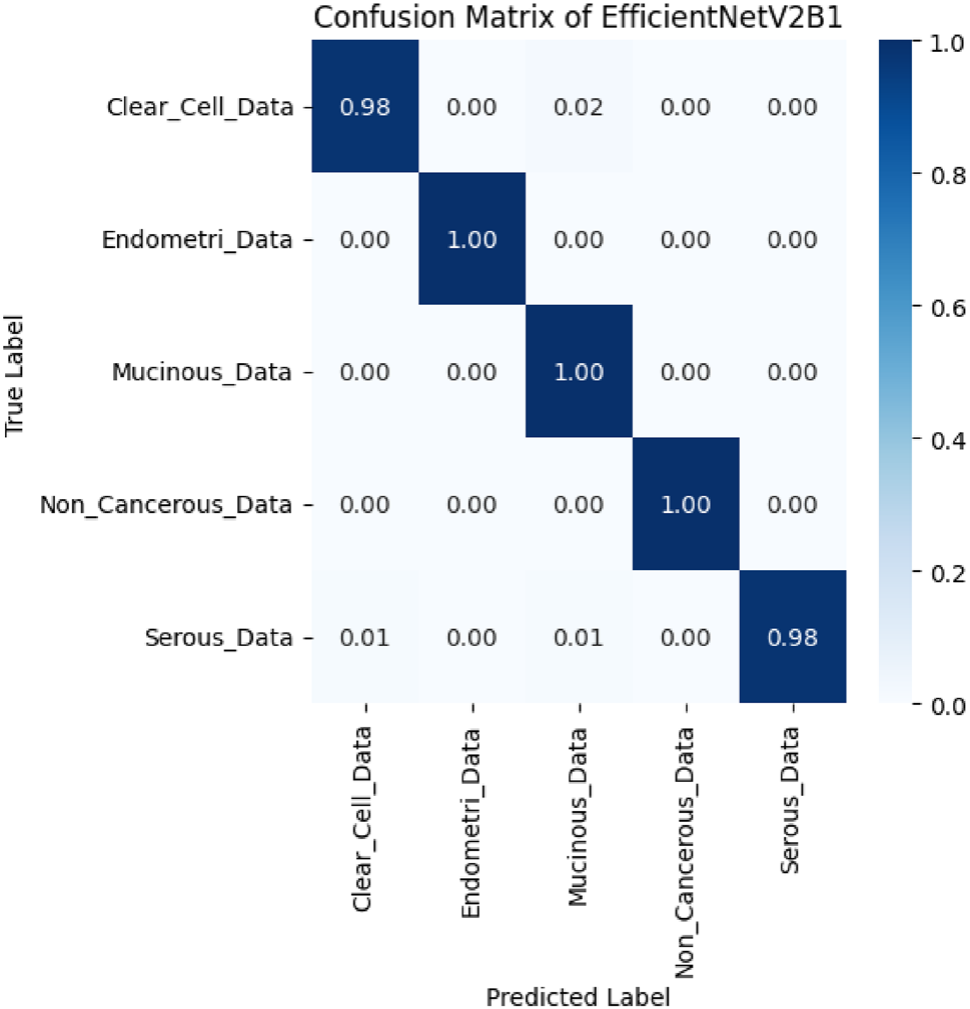
Confusion matrix of proposed OvCan-FIND model.

The proposed model OvCan-FIND performed better than the various base models, such as CNN models Inception V3, EfficientNet B1, EfficientNet B2, EfficientNet B3, ResNet152V2, MobileNet, MobileNetV2, VGG16, VGG19, and Xception, according to an analysis of the confusion matrix from Figure 3.

The OvCan-FIND model’s confusion matrix shows outstanding classification performance, with almost flawless accuracy across all ovarian cancer subtypes and non-cancerous cases. A high percentage of correctly identified instances is indicated by the diagonal values, which are near 1.00. This is especially true for Clear Cell, Endometri, Mucinous, and Non-Cancerous Data, which exhibit nearly no misclassification. The overall misclassification rate is very low, even while Serous Data shows very few errors (0.01 misclassified as Clear Cell or Mucinous). This demonstrates the model’s resilience, high accuracy, and dependability in differentiating between ovarian cancer subtypes, making it a viable tool for precise categorization of histopathological images. All of the models nevertheless yielded positive results as well as these models’ extensive performance is also notable^45–47^ but our OvCan-FIND model had the best overall performance.

**Fig. 4:**
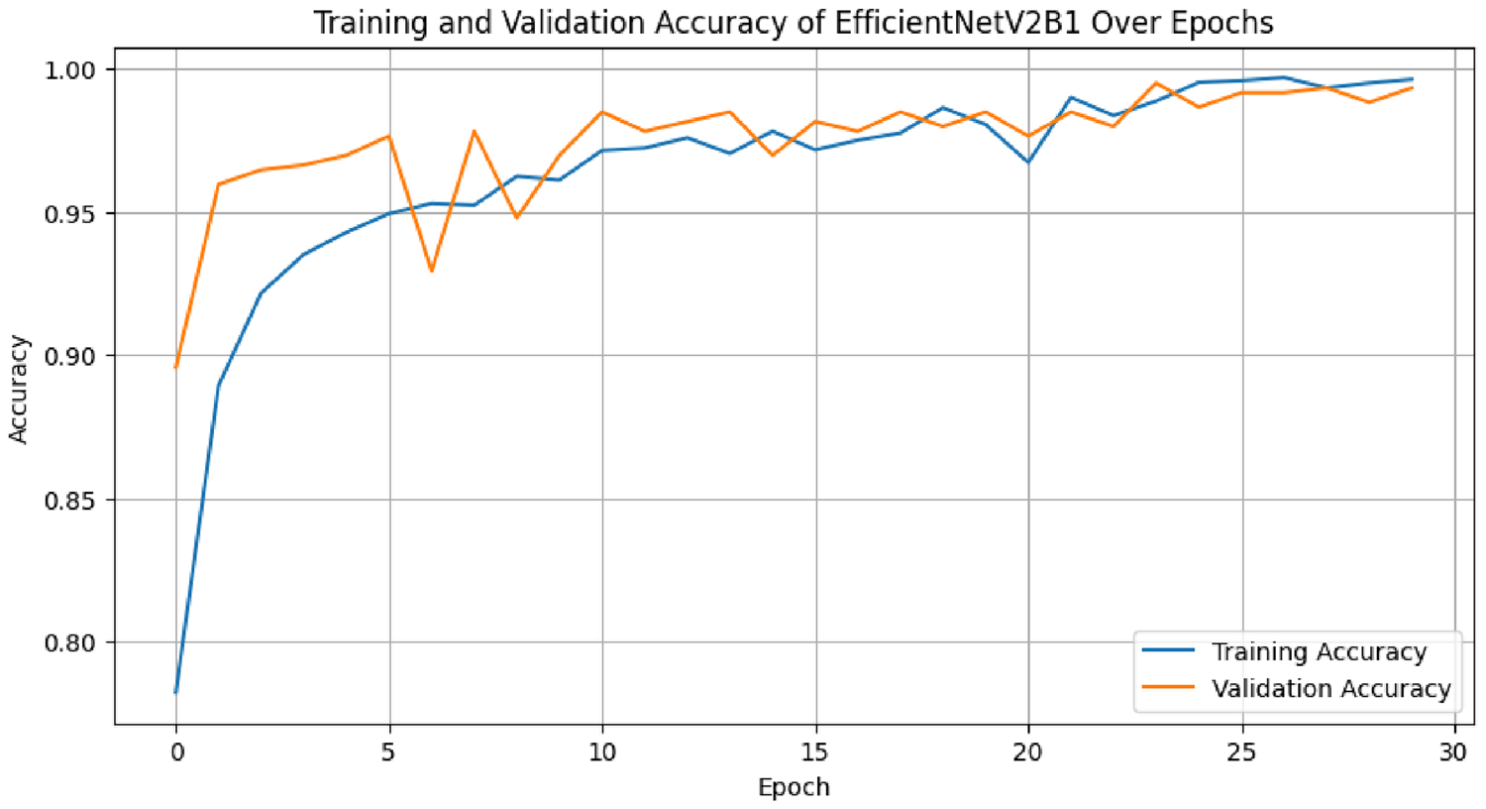
The training and validation accuracy graph of proposed OvCan-FIND model.

**Fig. 5:**
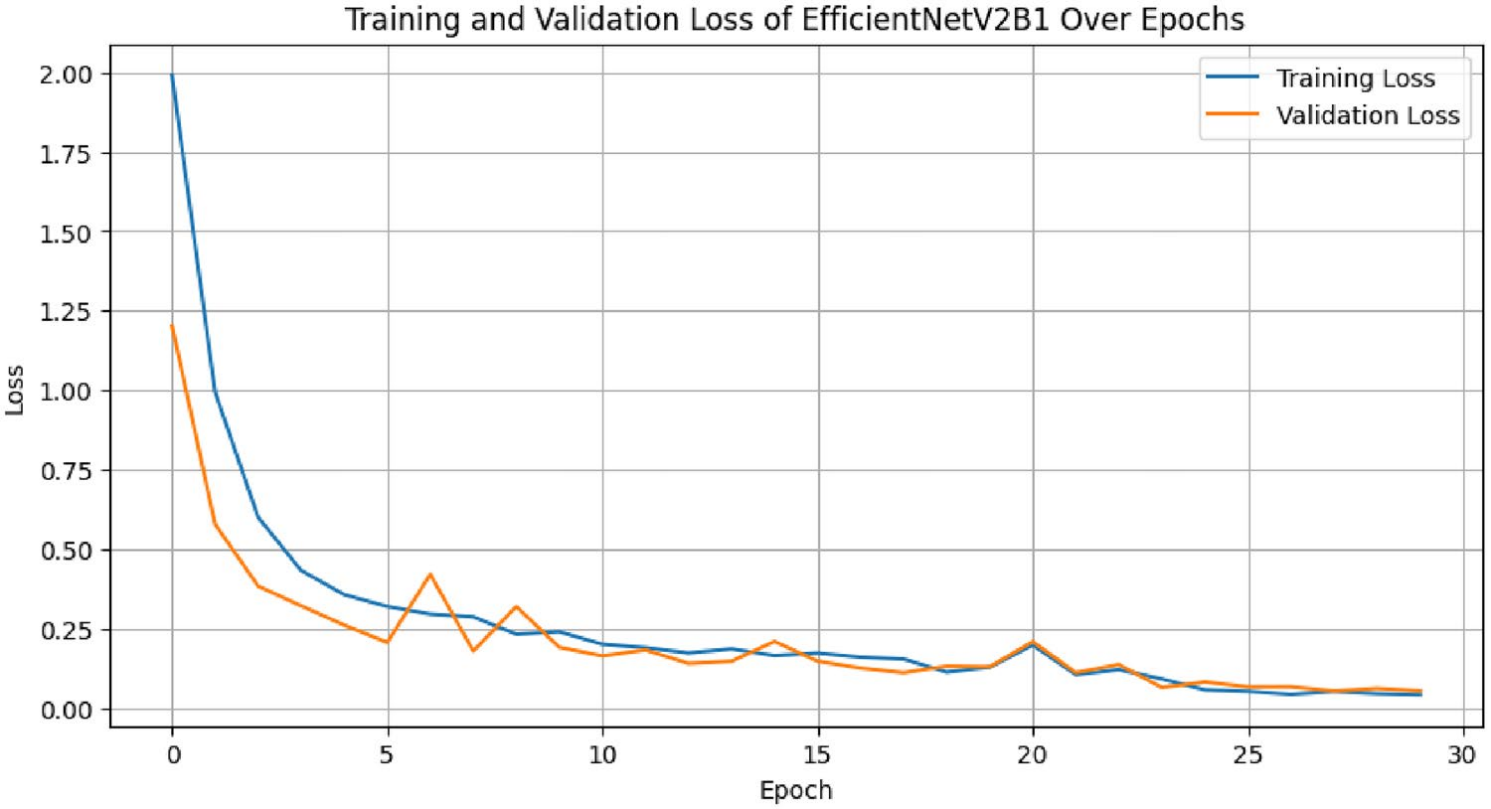
The training and validation loss graph of proposed OvCan-FIND model.

The diagonal blue hues in the confusion matrix diagram show the percentage of correctly predicted values that the model produced in comparison to the ground truth value. Figures 4 and 5 display the accuracy and loss curve outputs to further elucidate our model.

The OvCan-FIND model, when compared to base models that have been constructed, provides valuable insights into the effectiveness of deep learning architectures in medical imaging applications. By exceeding the base models in terms of accuracy, F1 score, recall, precision, and MCC (Matthews Correlation Coefficient) score, the suggested model displays its potential for better diagnostic accuracy. This superiority implies that, in comparison to its competitors, the OvCan-FIND model has better sensitivity and specificity because it can capture complex patterns and variables related to OC diagnosis. Moreover, the analysis of training and validation loss across epochs shows that the suggested model not only has better convergence but can also reduce overfitting, suggesting that it can robustly generalize to unknown data. These results highlight the potential contribution of customized deep learning models in improving the precision and dependability of OC diagnosis.

**Fig. 6:**
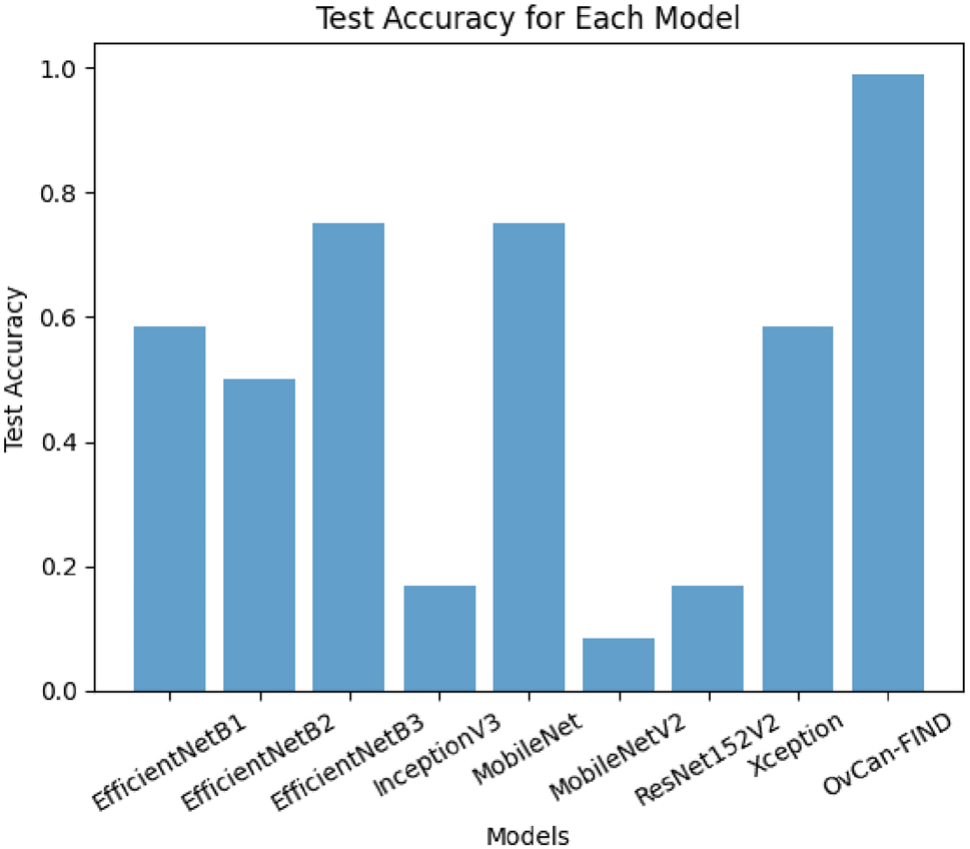
The accuracy graph for all models including OvCan-FIND.

**Fig. 7:**
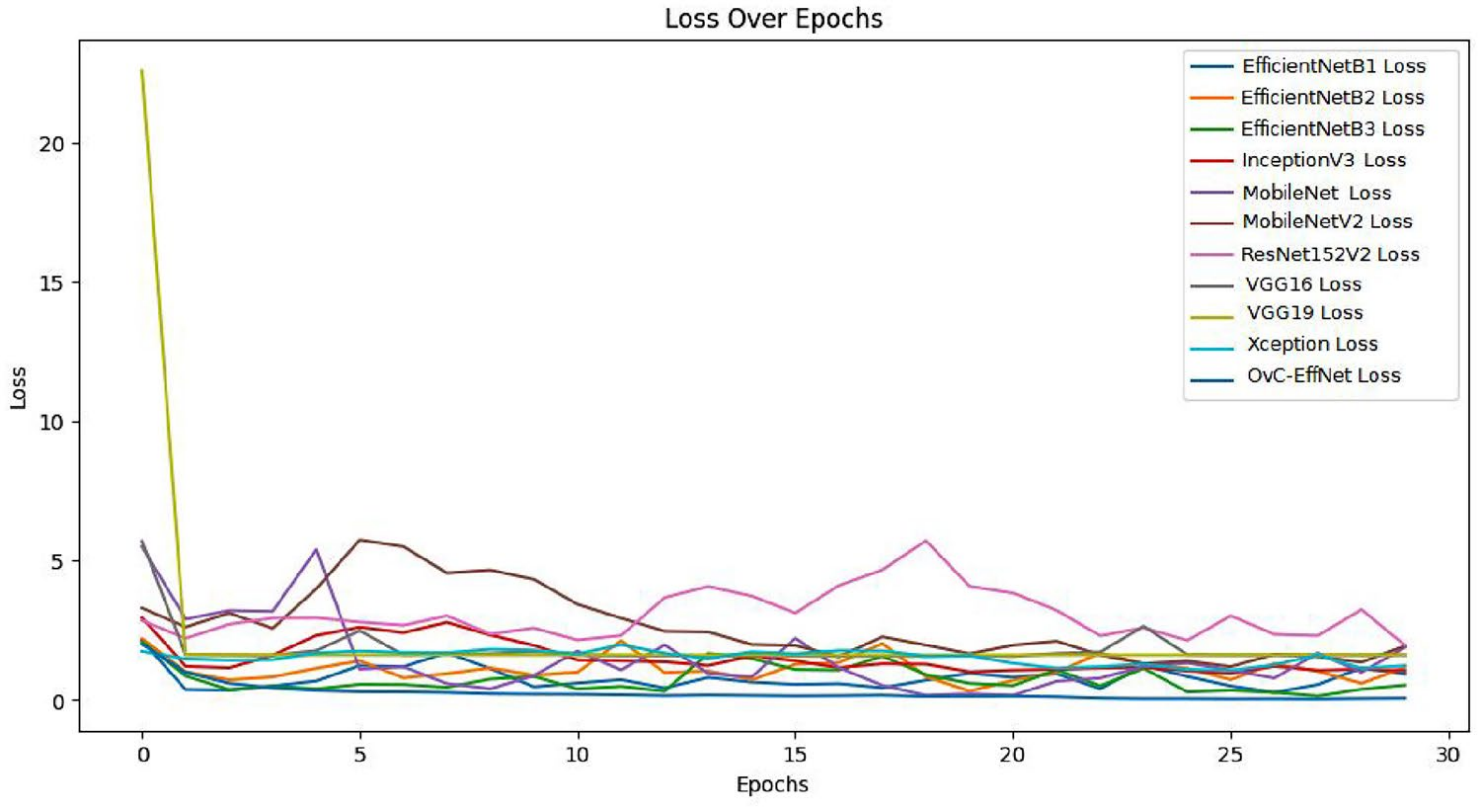
The training and validation loss graph for all models including OvCan-FIND.

**Fig. 8:**
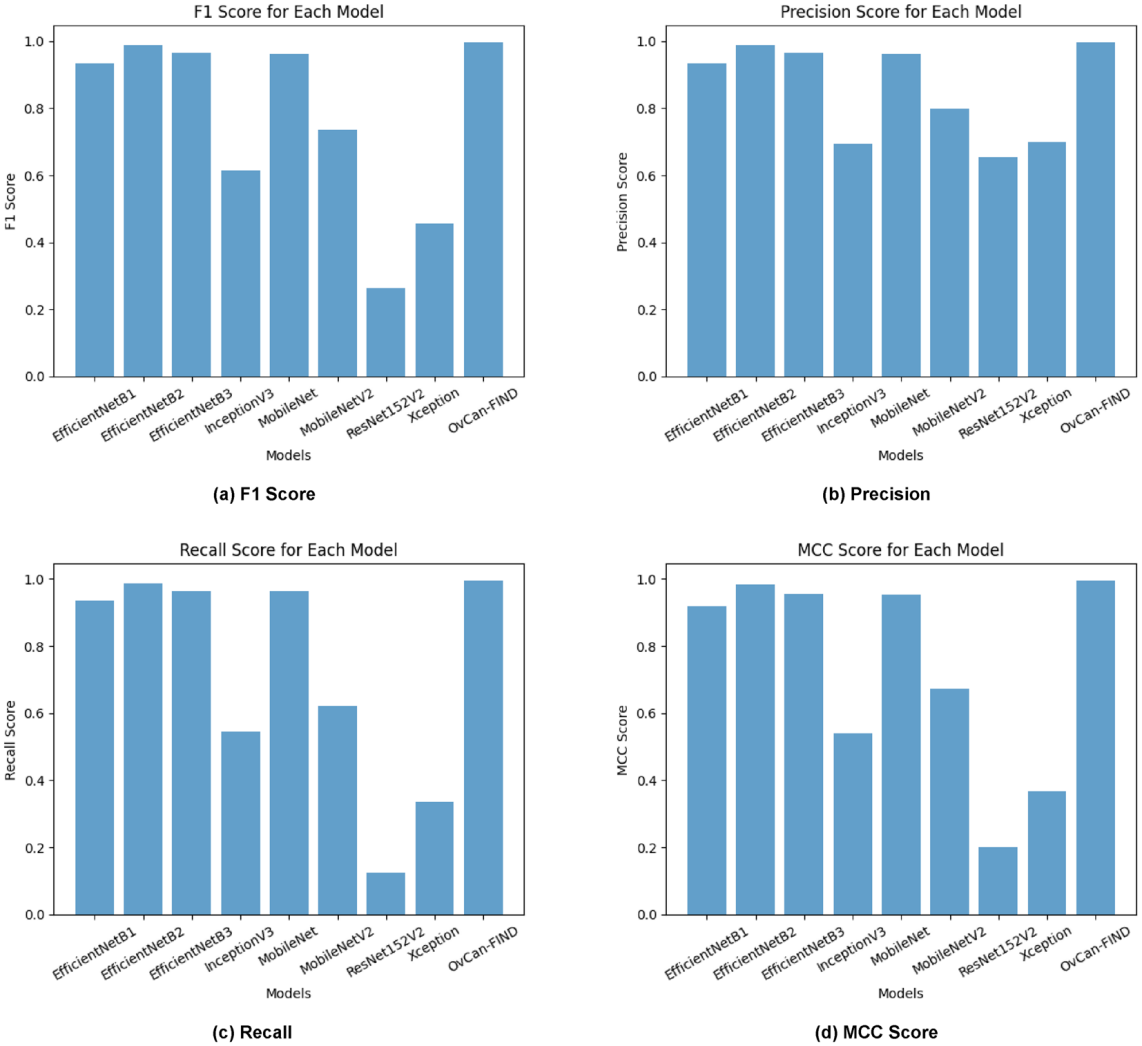
The comparison graph for all models including OvCan-FIND.

The OvCan-FIND model’s performance evaluation in comparison to a set of well-known base models highlights the importance of architectural design decisions in maximizing OC classification accuracy. This focused strategy not only produces better performance measures but also emphasizes how crucial domain-specific model creation is for applications involving medical imaging. The OvCan-FIND model’s success in outperforming its competitors in terms of accuracy, F1 score, recall, precision, and MCC score highlights the importance of continued research and innovation in improving these models for use in actual clinical settings. It also confirms the promise of deep learning methodologies in transforming cancer diagnosis. Figures 6, 7, 8 display the Accuracy, Loss, F1 score, Precision, Recall, and MCC graphs compared with other models, and outputs to further elucidate our model.

Table 6 provides an extensive analysis of model accuracies and performance indicators for image classification tasks. It includes the suggested OvCan-FIND model along with a number of well-known models from previous studies. Interestingly, the suggested model considerably exceeds the prior research, attaining an exceptional accuracy of 99.74%, whereas the latter works produced respectable accuracies ranging from 93% to 95.7%. Moreover, with a precision of 99.68%, recall of 99.56%, and an F1 score of 99%, the suggested model performs exceptionally well in terms of accuracy, recall, and F1 score. This demonstrates the OvCan-FIND model’s effectiveness and advances, establishing it as a top option for image classification problems.

**Table 6:** Model accuracy comparison with existing researches with proposed OvCan-FIND model.

**Author**	**Algorithm**	**Test accuracy**	**Precision**	**Recall**	**F1 score**
^7^	CNN	93%	93%	93%	94%
^27^	UNet	95.7%	-	-	-
^30^	YOLO v5	95%	99%	96%	97.5%
Proposed model	OvCan-FIND	99.74%	99.68%	99.56%	99%

## Discussion, challenges and future work

The OvCan-FIND model marks a significant stride in the field of medical imaging, with an accuracy rate that reaches an impressive 99.74% in OC classification. This achievement is not just a step forward but a leap that sets a new precedent, outstripping established CNN architectures like Inception V3 and ResNet152V2. The model’s performance is not only superior but also indicative of the transformative potential of advanced deep learning systems in healthcare diagnostics. The model’s architecture is a testament to thoughtful design, integrating layers such as Global average pooling 2 d, a Dense layer of 1024 units, Dropout, another Dense layer of 512 units, additional Dropout, and a final Dense layer of 5 units, with the Dense layers being accompanied by L2 Kernel Regularizers. These layers contribute significantly to the model’s enhanced performance metrics, including accuracy, recall, precision, and f1 score. The incorporation of the Adam optimizer, categorical cross-entropy loss function, and the ReLU and Softmax activation functions has been pivotal in the model’s success. These choices reflect a commitment to achieving a level of efficacy that sets the OvCan-FIND model apart. Furthermore, refining the EfficientNetV2 framework requires fewer computational resources when compared to other models, while delivering precise and robust results. This balance of efficiency and accuracy underscores the model’s potential as a significant contribution to the field of medical diagnostics.

The model’s adeptness in discerning intricate patterns and variables crucial for OC diagnosis underscores its potential to significantly enhance the precision and dependability of diagnostics. The groundbreaking results achieved by this model herald the advent of a new epoch in precision medicine, offering novel insights and opportunities for augmenting patient outcomes in the management of complex conditions such as OC. Consequently, the performance of the OvCan-FIND model exemplifies the profound impact that state-of-the-art deep learning models can have on medical imaging and healthcare diagnostics, charting a course towards heightened diagnostic accuracy and more tailored therapeutic approaches in the future. Furthermore as proven by the results and lack of a major computational overhead we can clearly see the divide between this model and other models, which puts into the limelight the strength of our model, with which it is able to achieve great results while maintaining efficiency and being less demanding on resources. While models like MobileNet may be more efficient, they still lack the ability to achieve the same performance that OvCan-FIND has achieved. But there is a lack of comparison with other research that has used the same dataset. This omission somewhat limits the ability to benchmark our findings against existing models and assess relative performance. Although the suggested OvCan-FIND model shows encouraging results in classifying ovarian cancer subtypes, there are a number of possible risks to its validity that need to be taken into account:

**Dataset Bias:** Because the model was trained and assessed using a particular dataset, it might not be as generalizable to data gathered from other organizations, scanners, or staining techniques. Validating the model on external, more varied datasets will be the main goal of future research.

**Class Imbalance:** Despite efforts to ensure that the training data was balanced, small differences in the distribution of classes may have an impact on the sensitivity of the model to under-represented subtypes.

**Overfitting Risk:** Given the excellent performance metrics noted, there is still a chance of overfitting even when data augmentation and regularization procedures are used. The robustness of the model would be further confirmed by additional validation on bigger, unseen cohorts.

**Lack of Explainability:** Explainable AI (XAI) methods like Grad-CAM and LIME were not used in this work. This restricts the model predictions’ interpretability, which is crucial for clinical implementation. This will be addressed in future research by incorporating XAI techniques.

**Evaluation Metrics:** Standard measures like accuracy, precision, recall, and F1-score were employed, but additional evaluation criteria, including uncertainty estimation and clinician-in-the-loop input would be needed for real-world clinical implementation.

By recognizing these risks, we hope to show the study’s shortcomings and suggest areas for further development.

## Conclusion

This study highlights the revolutionary potential of deep learning in medical imaging, namely in the use of sophisticated CNNs for the categorization of OC. With an astounding 99.74% accuracy rate, our suggested OvCan-FIND model marks a substantial breakthrough in OC detection. By showing how task-specific deep learning architectures can outperform conventional models in terms of accuracy and dependability, this work advances the discipline. By contrasting our model with well-known architectures like Xception, ResNet152V2, MobileNet, MobileNetV2, VGG16, VGG19, and Inception V3, we show how CNN-based classification has changed over time and position our model as a new standard for OC detection efficiency and accuracy. This study demonstrates the need for customized AI-driven methods in medical imaging and demonstrates how well-suited deep learning solutions may greatly enhance diagnostic performance.

Practically speaking, the OvCan-FIND model provides significant benefits in actual clinical settings. In order to improve patient outcomes and lower OC-related mortality rates, early detection, and prompt care are essential, and its high classification accuracy lowers diagnostic errors. Additionally, our approach guarantees improved generalizability across various histopathology datasets by tackling overfitting and performance instability, which makes it a feasible option for automated and objective OC classification. The increased dependability of AI-based diagnostic tools may reduce the workload for pathologists, reduce interpretive subjectivity, and speed up clinical judgment, all of which could result in a more effective and easily accessible cancer diagnosis.

This study has certain limitations in spite of these contributions. First, even though our model shows excellent accuracy, more testing is necessary to confirm its resilience across various imaging scenarios and patient demographics. Second, because of the dependence on histopathology pictures, real-time applicability in a clinical context is still unknown, which calls for future research to combine multimodal data, including genetic and radiological information, for a more thorough diagnosis approach. Last but not least, even while deep learning models are capable of achieving high classification accuracy, they frequently function as “black-box” systems, which restricts interpretability and clinician confidence in diagnoses made by AI. It will be necessary to address these issues in order to ensure smooth clinical adoption.

Future studies ought to look in a number of ways. First, using multi-modal fusion techniques that combine genomic, radiographic, and histopathological data may improve diagnosis precision and offer a more comprehensive understandings of the course and outcome of OC. Second, better decision-making and more clinician trust would result from research into explainable AI (XAI) strategies to increase model transparency. Finally, the accessibility and effect of the OvCan-FIND model would be greatly increased by creating computationally efficient and lightweight versions for real-time deployment in clinical settings, especially in contexts with restricted resources. These developments have the potential to further transform AI-powered cancer diagnostics and move oncology closer to precision and personalized medicine.

## Data Availability

The datasets analyzed for this study can be found in GitHub. This is available at: https://github.com/Anika-Saba-Ibte-Sum/Dataset
